# Glutamate/glutamine and neuronal integrity in adults with ADHD: a proton MRS study

**DOI:** 10.1038/tp.2014.11

**Published:** 2014-03-18

**Authors:** S Maltezos, J Horder, S Coghlan, C Skirrow, R O'Gorman, T J Lavender, M A Mendez, M Mehta, E Daly, K Xenitidis, E Paliokosta, D Spain, M Pitts, P Asherson, D J Lythgoe, G J Barker, D G Murphy

**Affiliations:** 1 Adult ADHD Service, The Maudsley Hospital, London, UK; 2 King's College London, Department of Forensic and Neurodevelopmental Sciences, Institute of Psychiatry, London, UK; 3 King's College London, MRC Social Genetic and Developmental Psychiatry Centre, Institute of Psychiatry, London, UK; 4 King's College London, Department of Neuroimaging, Institute of Psychiatry, London, UK; 5 Autism Assessment and Behavioural Genetics Clinic, South London and Maudsley NHS Foundation Trust, Bethlem Royal Hospital, Beckenham, UK; 6 Sackler Institute for Translational Neurodevelopment, Institute of Psychiatry, King's College London, London, UK

**Keywords:** ADHD, basal ganglia, dorsolateral prefrontal cortex, magnetic resonance spectroscopy, parietal lobe

## Abstract

There is increasing evidence that abnormalities in glutamate signalling may contribute to the pathophysiology of attention-deficit hyperactivity disorder (ADHD). Proton magnetic resonance spectroscopy ([1H]MRS) can be used to measure glutamate, and also its metabolite glutamine, *in vivo*. However, few studies have investigated glutamate in the brain of adults with ADHD naive to stimulant medication. Therefore, we used [1H]MRS to measure the combined signal of glutamate and glutamine (Glu+Gln; abbreviated as Glx) along with other neurometabolites such as creatine (Cr), N-acetylaspartate (NAA) and choline. Data were acquired from three brain regions, including two implicated in ADHD—the basal ganglia (caudate/striatum) and the dorsolateral prefrontal cortex (DLPFC)—and one ‘control' region—the medial parietal cortex. We compared 40 adults with ADHD, of whom 24 were naive for ADHD medication, whereas 16 were currently on stimulants, against 20 age, sex and IQ-matched healthy controls. We found that compared with controls, adult ADHD participants had a significantly lower concentration of Glx, Cr and NAA in the basal ganglia and Cr in the DLPFC, after correction for multiple comparisons. There were no differences between stimulant-treated and treatment-naive ADHD participants. In people with untreated ADHD, lower basal ganglia Glx was significantly associated with more severe symptoms of inattention. There were no significant differences in the parietal ‘control' region. We suggest that subcortical glutamate and glutamine have a modulatory role in ADHD adults; and that differences in glutamate–glutamine levels are not explained by use of stimulant medication.

## Introduction

Attention-deficit/hyperactivity disorder (ADHD) is a neurodevelopmental syndrome with childhood onset that affects ~5% of children and 2.5% of adults.^1^ The condition is characterised by pervasive, developmentally inappropriate levels of hyperactivity, impulsivity and inattention. ADHD leads to significant functional impairments and economic burden.^2^ ADHD in adults has historically been underdiagnosed and has received less research attention than childhood ADHD, but its importance is becoming more widely recognised.^3^

There is substantial evidence that individuals with ADHD have abnormalities in brain anatomy and function,^4^ and perhaps especially in dopamine neurotransmission^5,6^ within the prefrontal cortex (PFC) and the basal ganglia—areas known to be involved in both attention and the regulation of behaviour.

One line of evidence in favour of the ‘dopamine hypothesis' of ADHD comes from the efficacy of cathecholaminergic stimulant drugs, such as methylphenidate and amphetamine, in relieving ADHD symptoms. Stimulants enhance synaptic dopamine (and noradrenaline) levels by blocking dopamine reuptake transporters (DAT) and, in the case of amphetamine, by promoting dopamine release from presynaptic neurons.^7^

However, although stimulants are superior to placebo in improving ADHD symptoms in the short term,^8^ they are not effective in all cases. For instance, it is estimated that stimulants produce complete remission in ~30% of cases, with substantial but partial improvement in another 40%, and little or no benefit in 30%.^9^ Hence, deficient dopamine neurotransmission seems unlikely to be responsible for all cases of this heterogeneous disorder. In order to develop effective treatments for ADHD cases refractory to current medications, therefore, it may be necessary to look beyond dopamine.

Impaired glutamate signalling is another possible underlying abnormalities in (some cases of) ADHD, and an emerging treatment target.^10^ Glutamate is an amino acid involved in excitatory neurotransmission.^11^ Further, glutamate and dopamine interact, with glutamate modulating dopamine release in corticostriatal pathways and vice versa.^12^

There is some—albeit preliminary—evidence to suggest glutamatergic involvement in ADHD. For example, genetic studies have reported an association between ADHD and a number of glutamate receptor gene variants.^13^ Further, some animal models suggest that altered glutamate regulation of the dopamine system^14^ underpins the functional deficits associated with ADHD. Until relatively recently, however, it was impractical to measure human brain glutamate *in vivo*.

Proton magnetic resonance spectroscopy ([1H]MRS) can be used to quantify the combination of glutamate and its metabolic product glutamine (Glu+Gln, henceforth abbreviated Glx) in addition to other metabolites of potential clinical importance, such as choline (Cho) containing compounds, creatine (Cr) and phosphocreatine (Cr+PCr, hereafter abbreviated Cr), and *N*-acetylaspartate (NAA). It is generally accepted that Cr is involved in phosphate metabolism and reflects energy use and storage by neurons, whereas Cho is a measure of membrane synthesis and turnover, and NAA is a marker of neuronal density and/or mitochondrial function.^15^

Prior [1H]MRS studies of Glx and other metabolites in adults with ADHD^16,17^ (see the Discussion for details) have reported mixed findings. For example, some reported increases in Glx,^18^ whereas others found decreases.^19^ These studies were valuable first steps. However, they suffered from a number of limitations, for instance, most did not control for possible effects of prior use of ADHD medication (which has been shown to affect Glx and other metabolite levels.^20^)

Therefore, we investigated differences in brain glutamate/glutamine and other key neurometabolites in adults with ADHD and controls. In addition, we recruited both treatment-naive and stimulant-medicated ADHD individuals in order to allow us to ascertain whether any differences observed were associated with pharmacological confounds.

We acquired spectra from three [1H]MRS voxels, with the following locations: the caudate nucleus/striatum (basal ganglia), the dorsolateral PFC (DLPFC) and the medial parietal cortex. The caudate/striatum and the DLPFC were chosen on the grounds that abnormalities in both of these interconnected regions have been previously implicated in ADHD.^21^ Moreover, these two selected areas are known to be involved in processes relevant to the symptoms of ADHD: the caudate/striatum is responsible for the regulation of attention,^22,23^ whereas the DLPFC is a key part of the executive control network in the brain.^24,25^

We selected the third area, the medial parietal cortex, as a ‘control' region, as the structure and neurochemistry of this area have not previously been linked to ADHD. This was included as we wished to investigate whether any abnormalities seen in these disease-linked areas reflected specific local changes, or more widespread differences in metabolite levels.

## Materials and methods

### Participants

We included 60 adults: 40 with ADHD and 20 matched healthy controls (Table 1). The ADHD cohort was comprised of two groups—24 individuals who were stimulant naive and 16 individuals who were currently receiving stimulant (methylphenidate or dextroamphetamine) treatment (the prescribed dose of stimulants was not recorded). We included only right-handed participants in order to avoid possible effects of lateralisation, given that the [1H]MRS voxels in this study were unilateral.

ADHD participants were recruited from the Adult ADHD Service at the Maudsley Hospital in London, a specialist clinic providing diagnostic assessment and treatment for adults with ADHD referred from across the United Kingdom.

Diagnosis was performed by a consultant psychiatrist specialising in adult ADHD. Diagnosis was informed by pre-assessment questionnaires and by an in-depth clinical interview based around the ‘Conners Adult ADHD Diagnostic Interview DSM-IV', a structured clinical interview based on DSM-IV (Diagnostic and Statistical Manual, 4th Edition) criteria.^26^

Using Part II of ‘Conners Adult ADHD Diagnostic Interview DSM-IV', the ‘Diagnostic Criteria Interview for DSM-IV', information was gathered on current symptoms as well as on the age of onset, pervasiveness and level of impairment for each symptom present. Diagnosis was made if there was clear evidence of clinically significant impairment in more than two domains (social, academic or occupational functioning) in accordance with DSM-IV criteria. Therefore, all 40 participants with ADHD meet the DSM-IV diagnostic criteria for ADHD. In terms of clinical subtypes, these, 22 individuals meet the criteria for Combined Type ADHD and 18 individuals for inattentive type; none met criteria for hyperactive type. However, it is noteworthy that we have not conducted subgroup analyses, as we believe that correlational analysis using dimensional symptom measures is more appropriate.

The assessment also included obtaining a detailed account of past psychiatric history, developmental history, medical history and a mental state examination. Information regarding past psychiatric history was also provided by the referring clinician. Pre-assessment questionnaires included the Barkley Scales^27^, which provides both self- and informant assessment for (a) adulthood and (b) childhood symptoms.

Exclusion criteria for the ADHD group included the current presence of any other DSM-IV Axis I psychiatric disorder; an autistic spectrum disorder; a tic disorder; past or current substance abuse; significant medical or neurological illness affecting brain function; and the use of any psychotropic medication, other than stimulants, in the previous 6 months.

Although we included ADHD patients only if they had no current psychiatric comorbidity, 22 out of the 40 ADHD participants had a prior history of a psychiatric disorder(s) other than ADHD, including depression, anxiety and personality disorder. Furthermore, 14 ADHD patients had been exposed to psychotropic medication other than stimulants in the past, mainly antidepressants.

Healthy controls had no history of any psychiatric disorder, and were naive to psychoactive medication.

Ethical approval for this study was provided by South London and Maudsley/Institute of Psychiatry (SLaM/IoP) National Health Service Research Ethics Committee, study reference 1997/087. After complete description of the study to the subjects, written informed consent was obtained.

### Design and procedure

#### [1H]-MRS data acquisition

[1H]MRS data were acquired on a 1.5T GE HDx Magnetic Resonance Imaging (MRI) scanner (GE Medical Systems, Milwaukee, WI, USA) equipped with TwinSpeed gradients.

The scanning protocol included a structural MRI scan for the localisation of the spectroscopy voxels in each participant, namely a three-dimensional fast inversion-recovery prepared spoiled gradient echo (SPGR) acquisition with number of slices=146, slice thickness=1.2 mm, inversion time=300 ms, repetition time=11 ms, echo time=5 ms, field of view=310 mm, flip angle=18°, matrix=256 × 160 over a 310 × 194 mm field of view, giving 1.20 × 1.20 × 1.20 mm^3^ voxels. Full Scale IQ was measured using the Wechsler Abbreviated Scale of Intelligence.

Three single voxel [1H]MRS spectra were then acquired, using a point-resolved spectroscopy sequence. Point-resolved spectroscopy parameters were: repetition time=3000 ms and echo time=30 ms. A total of 72 (for the parietal) or 104 (for basal ganglia and DLPFC voxels) repeat observations were averaged to produce the final spectrum. In addition to this, eight observations without water suppression were averaged and used for absolute water scaling.

**Figure 1:**
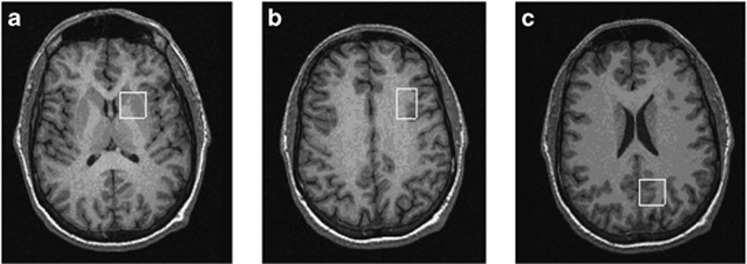
Location of voxels of interest (VOIs). VOIs were positioned in (**a**) left basal ganglia (20x20 × 15 mm^3^) to include the head of the caudate, putamen and internal capsule; (**b**) left dorsolateral prefrontal cortex (16x24 × 20 mm^3^); and (**c**) left medial parietal lobe (20x20 × 20 mm^3^).

The first voxel (20x20 × 20 mm^3^) was placed in the left medial parietal lobe. The second voxel (20x20 × 15 mm^3^) was positioned in left basal ganglia to include parts of the head of the caudate, the anterior putamen and the internal capsule. The last voxel (16x24 × 20 mm^3^) was placed in the left DLPFC. See Figure 1 for an illustration of the location of these voxels.

Standardised placement procedures were used to ensure the anatomical comparability of the voxels between participants. For example, for the basal ganglia voxel, on the structural MRI, the axial slice where the width of the putamen in the right–left direction was widest was selected. The centre of the voxel was placed on this slice, such that the anterior edge lay on the anterior margin of the head of the caudate and the medial edge lay on the border of the lateral ventricles. These methods have been described in detail previously.^28, 29, 30^

The order of image acquisition was fixed as follows: (1) structural MRI, (2) parietal voxel, (3) basal ganglia voxel, (4) DLPFC voxel and (5) phantom. The total scan time was ~45 min.

#### Data processing

[1H]MRS spectra were processed using LCModel software version 6–1–0 (Stephen Provencher Inc., Oakville, ON, Canada). LCModel uses a linear combination of model spectra of metabolite solutions *in vitro* to analyse the major resonances of *in vivo* spectra. In this case, a basis set of alanine, aspartate, Cr, gamma-aminobutyric acid, glutamine, glutamate, glycerophosphocholine, *myo*-inositol, lactate, NAA, N-acetyl-aspartylglutamate (but note, we here report NAA+N-acetyl-aspartylglutamate combined as ‘NAA' for simplicity), scyllo-inositol and taurine, together with a baseline function were used for analysis.

#### Calculation of absolute metabolite concentrations

**Figure 2:**
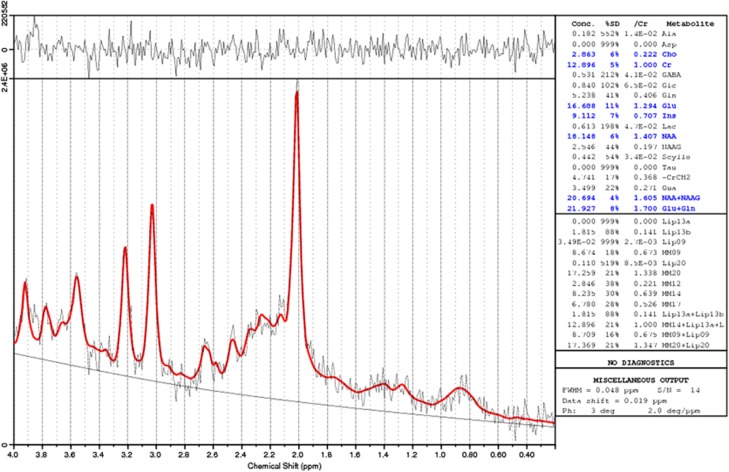
Example of an 1.5 Tesla proton magnetic resonance spectroscopy ([1H]MRS) spectrum showing LCModel 6–1–0 fit. This spectrum was acquired from the dorsolateral prefrontal cortex voxel (DLPFC).

Metabolite concentrations for NAA, Cr, Glx and Cho were calculated, in institutional units, as follows. Raw estimates (LCModel output) were corrected by reference to calibration data from a phantom containing an aqueous solution of known NAA concentration. A phantom [1H]MRS spectrum was acquired at the end of each scanning session. The ratio of the known concentration of NAA in the phantom to the observed NAA phantom concentration was used to derive a correction factor that was applied to calibrate the metabolite concentrations into molar units. This served to control for possible scanner ‘drift' in raw [1H]MRS estimates. See Figure 2 for an example LCModel fit of one of the spectra included in this study.

To guard against partial volume confounds (group differences in proportions of grey matter, white matter and cerebrospinal fluid (CSF) in the [1H]MRS voxels), we also corrected the metabolite concentrations for voxel composition. We first determined the percentage of grey matter, white matter and CSF within each [1H]MRS voxel for each participant by segmenting the SPGR structural volume, using an automated procedure, *spm_segment*, part of the Statistical Parametric Mapping software package (http://www.fil.ion.ucl.ac.uk/spm/).

The position of each individual [1H]MRS voxel relative to the corresponding structural image was determined using positional coordinates embedded in the spectra data files. The % grey, white and CSF composition of each voxel was then calculated automatically from the segmented images using in-house software. Finally, metabolite concentrations were corrected for voxel % (CSF) by multiplying values by an individual correction factor=1/(1−Proportion_CSF_), where Proportion_CSF_ could range from 0 to 1.

In summary, Metabolite_corrected_=Metabolite_raw_ × (PhantomNAA_known_/PhantomNAA_observed_) × (1/(1–Proportion_CSF_)).

#### Statistical analysis

All statistical analysis was performed using SPSS 15.0 (SPSS, Chicago, IL, USA).

First, participant demographic information for the groups was compared using *χ*^2^ analyses and one-way analyses of variance across the three participant groups, namely, stimulant-naive ADHD participants, medicated ADHD participants and healthy controls.

Voxel tissue composition was compared using independent samples *t*-tests comparing participants with ADHD (*n*=40) with the healthy controls (*n*=20) for each of (i) grey matter, (ii) white matter and (iii) CSF in each voxel. We further compared the two ADHD groups, ADHD naive and ADHD medicated, with independent samples *t*-tests.

The primary [1H]MRS analysis was a series of independent samples *t*-tests comparing all participants with ADHD (*n*=40) to the matched healthy controls (*n*=20), for each corrected metabolite value (four), in each voxel (three). As this analysis involved multiple (12) comparisons, we performed a Bonferroni correction raising the significance threshold for each comparison from *P*=0.05 to *P*=0.004. We report results both before and after the Bonferroni correction.

In order to test for the possibility of medication effects on metabolites, we compared the two ADHD groups, ADHD naive and ADHD medicated, with independent samples *t*-tests. We further compared metabolite values in those ADHD participants who had never been prescribed any prescribed psychoactive medication in the past to healthy controls to verify the absence of any possible medication confounds.

We explored possible correlations between metabolite abnormalities and symptom severity in stimulant-naive participants with ADHD. We calculated bivariate Pearson's correlations between those metabolite concentrations that were significantly (at *P*<0.05 Bonferroni corrected) different from controls, and self-reported total Barkley Scale Inattention and Hyperactivity symptom scores.

Finally, to verify that our results were not artefacts of differences in the quality of the spectra, we examined group differences in % Cramer–Rao Lower Bounds (%CRLBs) for each metabolite for each voxel. Higher %CRLBs indicate greater uncertainty in metabolite estimation.

## Results

### Participant group characteristics

Participant characteristics are given in Table 1. There were no significant differences in age or full-scale IQ (*P*>0.05) across the participant groups. The two patient groups did not differ in mean symptom severity. The mean duration of stimulant treatment in the ADHD-medicated group (*n*=16) was 86 weeks at the time of scanning (s.d.=180, range=1–624 weeks).

### Primary analysis

#### Basal ganglia

**Figure 3:**
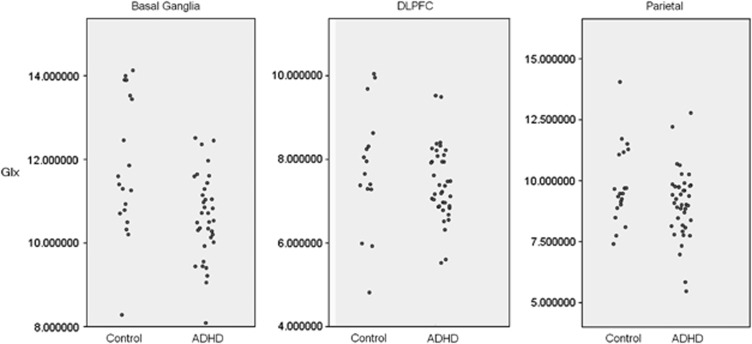
Comparison of glutamate/glutamine (Glx) in basal ganglia, dorsolateral prefrontal cortex (DLPFC) and parietal cortex voxels in healthy control participants and participants with attention-deficit hyperactivity disorder (ADHD). All concentrations are in institutional absolute units.

ADHD participants had a significantly lower concentration of Glx, Cho, Cr and NAA with all except Cho surviving Bonferroni correction for multiple comparisons (Table 2 and Figure 3).

#### DLPFC

Individuals with ADHD had significantly lower Glx, Cr and NAA; however, only the difference in Cr survived Bonferroni correction.

#### Parietal lobe

Only NAA was altered in ADHD in this voxel; this did not survive Bonferroni correction.

### Secondary analyses

#### Voxel composition

There were no significant differences between the ADHD and control groups in the mean tissue composition of the [1H]MRS voxels for grey matter, white matter or CSF (Table 3) in either the basal ganglia or DLPFC voxels. In the parietal voxel, there was a small but significant (*P*<0.05) difference in % CSF only. Nevertheless, because all metabolite concentrations were corrected for voxel % CSF (see Materials and methods), this is unlikely to have affected our results. There were no differences between the ADHD-naive and ADHD-medicated groups in any region (*P*>0.35).

#### Spectrum quality

Across the three voxels and the four metabolites, analyses of variance revealed no significant group differences in % CRLB, except in Glx in the DLPFC voxel where they differed significantly (*P*=0.004); here, the healthy controls had higher %CRLB, indicating worse data quality. As this was the only instance in which quality differed, and as the ADHD patients (the group with lower estimates) had better quality than the controls in this case, we are confident that our findings of reduced concentrations of various metabolites in ADHD patients are not an artefact of group differences in data quality leading to globally biased measures. See Table 4 for details.

#### Effect of stimulant medication

There were no significant differences between ADHD-naive and ADHD-medicated participants in any metabolite value, in any region (all *P*>0.139), even without correcting for multiple comparisons (Table 2). We further compared metabolite values in ADHD participants with no prior or current use of any prescribed psychoactive medication (*n*=15), including stimulants or any other drug classes, with the healthy controls (*n*=20). The basal ganglia Glx, Cr and NAA, and DLPFC Cr, were still significantly reduced (*P*<0.05 uncorrected), confirming most findings despite a reduced sample size.

#### Metabolites and symptoms

**Figure 4:**
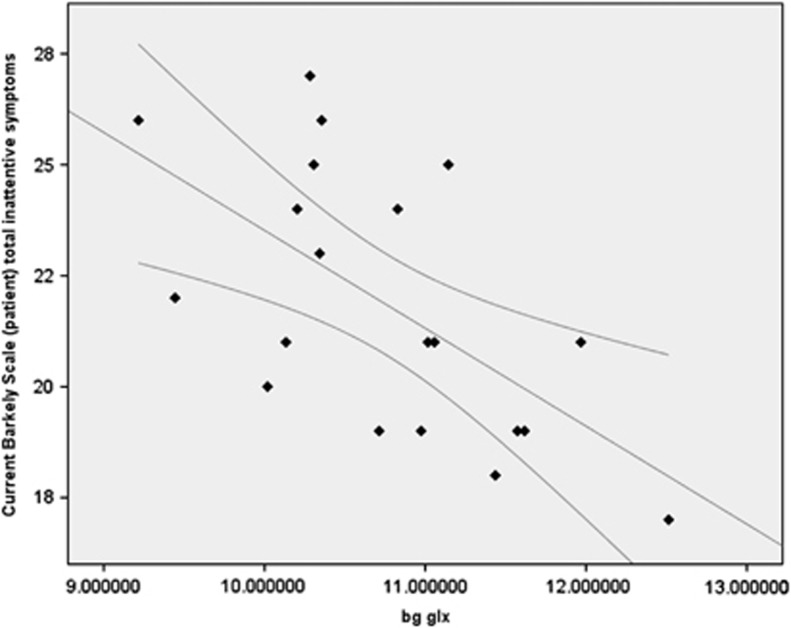
Absolute concentration of glutamate/glutamine (Glx) in the basal ganglia voxel was negatively correlated with total Barkley Scale Inattention score (*r*=−0.610, *P*=0.004), with lower (more abnormal) Glx concentrations associated with more severe symptoms of inattention. Scatterplot shows data points with linear correlation and 95% mean confidence intervals. Glx concentration is in institutional units.

An exploratory analysis of symptom dimensions in stimulant-naive ADHD participants revealed that the concentration of Glx in the basal ganglia voxel was negatively correlated with total Barkley Scale Inattention score (*n*=24, *r*= −0.610, *P*=0.004), with lower (more abnormal) Glx concentrations associated with more severe symptoms of inattention (Figure 4). However, we observed no significant correlation between basal ganglia Glx and hyperactive symptoms (*r*=0.247, *P*=0.294) or between basal ganglia Cr, basal ganglia NAA and DLPFC Cr, and any other symptoms (all *P*>0.13).

#### Past psychiatric comorbidity

As specified above, 22 of the 40 ADHD patients reported at least one past psychiatric comorbidity (though none had a current comorbidity), whereas none of the controls did. To verify that psychiatric history did not influence our MRS findings, we ran a series of independent group *t*-tests comparing ADHD-past-comorbid vs ADHD-never-comorbid patients for each of the four metabolites in the three regions (*n*=40). There was no significant difference on any MRS metabolite in any region (12 tests; all *P*-values >0.141 uncorrected).

## Discussion

We report significantly reduced concentrations of glutamate/glutamine (Glx) in the caudate/putamen of adults with ADHD, compared with matched healthy controls as measured using [1H]MRS. We also observed reductions in Cr and NAA in this region in the ADHD group, as well as reduced Cr in the DLPFC.

It is likely that these reductions represent a primary correlate of ADHD, rather than being secondary to medication effects, because we observed no differences between ADHD patients who were receiving stimulant medication compared with those who were medication naive. Also, our findings are not accounted for by differences in voxel grey/white matter proportion or by participant age, sex and IQ.

Furthermore, we are confident that our finding of reduced concentrations of certain metabolites in ADHD does not reflect group differences in data quality. Those metabolite levels that had differences that survived Bonferroni correction did not differ in precision as estimated with %CRLB, which suggests that our results cannot be explained as a result of, for instance, degraded spectra due to increased within-scanner motion in the ADHD group reflecting hyperkinesis or ‘fidgeting'.

Our data therefore add to an emerging literature on neurochemical abnormalities in adults with ADHD. Our finding of reduced Glx in the caudate/putamen is complemented by other studies that reported reduced Glx in the anterior cingulate cortex and the medial PFC.^17,19^ However, this reduction probably does not affect whole brain, because we observed no significant differences in the DLPFC or the parietal ‘control' region in the current study, and increased glutamate has previously been reported in the cerebellum of ADHD adults.^18^

Therefore, reduced Glx in adults with ADHD is not a brain-wide phenomenon, but seems to be affecting particular neural circuits. The reduction in Glx may also be age dependent, as studies in ADHD children have reported increased Glx, including significant increases in the frontal cortex^31, 32, 33^ and significant^34^ or nearly significant^31^ increases in the striatum (although see Yeo *et al.*^35^ and Sun *et al.*^36^).

Our results thus add to emerging evidence that ADHD is associated with glutamate pathway abnormalities at least in some cases. This evidence includes the discovery of rare deleterious variants in metabotropic glutamate receptors and other related genes in a minority of ADHD cases^37^ and also from animal models.^38^ This evidence has led to the development of glutamate modulatory drugs as potential treatments for ADHD, including the novel AMPA receptor-positive modulator Org26576, which was recently shown to be effective in a preliminary trial.^10^ Our data suggest that, at least in adults, the caudate/striatum may be the most important region implicated in glutamate dysfunction in ADHD.

As well as reduced Glx, we found reduced NAA and Cr in the caudate and putamen, and reduced Cr in the DLPFC. This contrasts with two prior [1H]MRS studies of adult ADHD that found reduced NAA in the DLPFC,^16^ and no differences in Glx^16^ or increased Glx:Cr ratio^39^ (with no absolute Glx reported) in the caudate and striatum. However, these two studies were small (*n*=5 (ref. 16) and 10 (ref. 39) ADHD patients, respectively) and did not include a ‘control' region. Thus, it is not clear that they possessed the statistical power to detect the effects were observed.

The metabolic abnormalities we detected, primarily in the caudate/putamen, may help to explain some of the core symptoms of ADHD, because this brain area is known to have a key role in the regulation of attention. For example, the basal ganglia are activated during the performance of set-shifting, reversal learning and task-switching paradigms,^22^ and in modulating the top-down influence of the PFC during shifts of attention.^23^

Consistent with its role in the flexible control of attention, we observed a significant correlation between caudate/striatum Glx and the Barkley Scale measure of the severity of attentional deficits, with lower, that is, more abnormal Glx being associated with worse attentional impairment, but only in individuals who were unmedicated. In medicated participants, although Glx was lowered to the same extent, there was no correlation with inattention, raising the possibility that dopaminergic stimulants may compensate for, but not reverse, an underlying glutamatergic abnormality. Although, given that this correlation is an exploratory *post hoc* finding, it should be treated with caution.

What might be the origin of the reduction in Glx that we observed in adult ADHD? It could be a primary deficit. Alternatively, it may be secondary to abnormalities in other neurotransmitter systems that we were unable to measure in this study. Dopamine pathways, for instance, richly innervate the caudate and putamen, and they area key site of dopamine–glutamate interactions.^40^

Dopamine is also known to regulate glutamatergic cell firing and vice versa.^40^ As ADHD is classically linked to a dopamine deficit,^5,6^ it is possible that, at least in some cases of the disorder, this is associated with changes in Glx. This could also explain the findings of reduced Glx in the medial PFC and anterior cingulate cortex (see above), as these areas are, similar to the basal ganglia, major targets of dopamine.

Another possibility is that differences in Glx might be secondary to abnormal cellular metabolism. For instance, we also observed significantly reduced concentrations of Cr and NAA, in the basal ganglia voxel and of Cr in the DLPFC. This could indicate decreased neuronal metabolism and energy use, as both Cr/PCr and N-acetylaspartate ([1H]MRS Cr and NAA peaks, respectively) are involved in neuronal energy metabolism.^41,42^ Such an energy deficit would be in line with glucose metabolism studies in adults with ADHD that have shown reduction of metabolism in both hemispheres.^43^

However, an alternative possible basis for reductions in Cr and NAA is reduced neuronal density. We did not observe differences in the voxel compositions of grey and white matter in the basal ganglia and DLPFC, showing that these metabolite differences were not simply partial volume effects caused by different proportions of brain tissue. However, it is possible that there were fewer (glutamatergic) neurons per unit of volume, leading to reduced estimates of these neuronal metabolites. Unfortunately, we are not aware of any studies that have examined neuronal density in the basal ganglia in ADHD.

Our study has a number of limitations. We obtained [1H]MRS data at a relatively low field strength of 1.5 Tesla. This could have limited the signal-to-noise ratio of our measures and, hence, it is possible that there exist further metabolite differences beyond the ones that we detected. Furthermore, we are unable to say whether the reliability of this 1.5T MRS compares with that obtained at higher field strengths. However, we do not believe that this undermines the robustness of those differences we did observe, rather it suggests that those differences we found are likely to be meaningful, as they can be detected even at 1.5 Tesla.

Another limitation arising from our use of 1.5 Tesla [1H]MRS is that we were unable to distinguish between the compounds that contribute to the ‘Glx' signal, that is, glutamate and glutamine, as their proton resonance signals overlap at low field strengths. Although glutamine is not directly involved in neurotransmission, it serves as an intermediate in the recycling of glutamate after its release and reuptake. Therefore, the glutamate:glutamine ratio may provide important information about the rate of glutamate synthesis and turnover.^44^ To understand the nature of the Glx abnormalities seen in ADHD, future work should therefore use higher field strength MRS (3 or 7 Tesla) to measure glutamate and glutamine separately.

A further issue is that we were not able to directly investigate the effects of medication on neural metabolites in ADHD, because patients were not examined before and after their treatment. We do not think that medication effects influenced our results, because we found no differences between adults with ADHD currently taking medication as compared with medication-naive adults. It is possible, however, that effects on neurometabolites might be dose-dependent; however, we did not have access to information on the prescribed dosage in those individuals who were taking stimulants. Therefore, an additional limitation of our study is that we were unable to examine possible dose-related effects and we cannot exclude the possibility that treatment with high doses of stimulants does exert an effect.

Finally, although our study included a ‘control' region, the medial parietal cortex, on the basis that it has not classically been implicated in ADHD, some recent functional imaging studies have reported alterations in activation in this area in ADHD.^45^ Thus, it may not be unrelated to the pathophysiology of the disorder. However, as we did not observe any (corrected) significant neurochemical differences in this area, the parietal data nonetheless support the regional specificity of the other findings.

In conclusion, adults with ADHD have regionally specific reductions in glutamate/glutamine and the neuronal energy metabolites NAA and Cr. Also reduction in striatal glutamate/glutamine is associated with more severe symptoms of inattention in medication-naive individuals. Glutamate may be a tractable treatment target is some adults with ADHD.

## Figures and Tables

**Table 1:** Participant characteristics

	*Controls (*n*=20)*	*ADHD (*n*=40)*	
		*Medication* *naive (*n*=24)*	*Medicated (*n*=16)*
Age	33 (7)	29 (9)	33 (5)
Full-scale IQ	116 (22)	104 (14)	114 (16)
Barkley (current, self-report) Total Inattentive Score	NA	22.3 (3.1)	22.6 (3.7)
Barkley (current, self-report) Total Hyperactive Score	NA	18.0 (5.3)	18.3 (7.0)
Sex (male/female)	15/5	17/7	14/2
Stimulant medication	Never	Never	Current methylphenidate (*n*=15); current dextroamphetamine (*n*=1)
Duration of stimulant treatment, weeks	NA	NA	86 (180)

Abbreviations: ADHD, attention-deficit hyperactivity disorder; NA, not applicable.

All data are given as group means (with s.d. in brackets). Note that data on ADHD participants are presented separately for the Medication naive and Medicated subgroups.

**Table 2:** [1H]MRS data

	*Healthy control*	*ADHD*			P*1-value*	P*2-value*
		*Total*	*Medicated*	*Not medicated*		
*Basal ganglia*						
Cho	1.27 (0.27)	1.12 (0.2)	1.13 (0.18)	1.11 (0.59)	0.043*	0.966
Cr	6.18 (1.32)	5.19 (0.56)	5.21 (0.58)	5.18 (0.56)	0.001**	0.996
*N*AA	6.48 (1.34)	5.27 (0.75)	5.22 (0.48)	5.31 (0.90)	0.000**	0.966
Glx	11.94 (1.7)	10.61 (1.0)	10.4 (1.21)	10.7 (0.82)	0.002**	0.727
*Dorsolateral prefrontal cortex*						
Cho	1.24 (0.3)	1.09 (0.12)	1.06 (0.14)	1.11 (0.10)	0.040*	0.701
Cr	4.37 (0.75)	3.85 (0.29)	3.85 (0.36)	3.86 (0.25)	0.001**	0.999
NAA	6.38 (1.18)	5.71 (0.44)	5.69 (0.53)	5.72 (0.39)	0.006*	0.994
Glx	7.84 (1.43)	7.42 (0.87)	7.44 (1.15)	7.41 (0.65)	0.261	0.994
*Parietal cortex*						
Cho	1.03 (0.23)	0.95 (0.17)	0.91 (0.16)	0.97 (0.17)	0.234	0.604
Cr	4.86 (0.64)	4.67 (0.57)	4.61 (0.55)	4.71 (0.58)	0.365	0.842
NAA	7.14 (1.03)	6.56 (0.54 )	6.44 (0.56)	6.64 (0.53)	0.022*	0.699
Glx	10.13 (1.74)	9.03 (1.40)	9.45 (1.44)	8.76 (1.33)	0.060	0.342

Abbreviations: [1H]MRS, proton magnetic resonance spectroscopy; ADHD, attention-deficit hyperactivity disorder; Cho, choline; Cr, creatine plus phosphocreatine; Glx, glutamate and glutamine; NAA, N-acetylaspartate.

Data are given as mean (s.d.); *significant at *P*<0.05, uncorrected for multiple comparisons; **significant at *P*<0.05 after conservative Bonferroni correction for multiple (12) comparisons.

*P*1, controls vs ADHD (both stimulant naive and medicated) *t*-test; *P*2, ADHD naive vs ADHD medicated.

**Table 3:** Voxel composition by group

	*Healthy control*	*ADHD*			P*1-value*	P*2-value*
		*Total*	*Medicated*	*Not medicated*		
*Basal ganglia*						
Grey	0.70 (0.12)	0.72 (0.083)	0.71 (0.06)	0.73 (0.098)	0.406	0.542
White	0.37 (0.12)	0.33 (0.08)	0.34 (0.06)	0.33 (0.09)	0.221	0.690
CSF	0.008 (0.01)	0.007 (0.01)	0.007 (0.01)	0.008 (0.01)	0.886	0.906
*Dorsolateral prefrontal cortex*						
Grey	0.28 (0.10)	0.25 (0.06)	0.24 (0.04)	0.26 (0.07)	0.156	0.453
White	0.75 (0.10)	0.78 (0.07)	0.78 (0.05)	0.77 (0.08)	0.297	0.660
CSF	0.017 (0.01)	0.014 (0.01)	0.014 (0.01)	0.014 (0.01)	0.340	0.959
*Parietal cortex*						
Grey	0.45 (0.05)	0.47 (0.07)	0.48 (0.08)	0.47 (0.06)	0.184	0.690
White	0.51 (0.08)	0.5 (0.08)	0.48 (0.09)	0.51 (0.06)	0.591	0.373
CSF	0.09 (0.04)	0.069 (0.03)	0.074 (0.03)	0.066 (0.03)	0.022*	0.388

Abbreviations: ADHD, attention-deficit hyperactivity disorder; CSF, cerebrospinal fluid. Data are given as mean (s.d.). Values represent estimated mean proportion of voxel (range 0–1). *P*1, controls vs ADHD (both stimulant naive and medicated) *t*-test; *P*2, ADHD naive vs ADHD medicated. These showed no significant differences except in parietal cortex % CSF (*P*<0.05) that was significantly higher in the healthy controls compared with the ADHD groups, with no differences between the medicated and medication naive ADHD groups. * indicates that the comparison was significant at *P*<0.05.

**Table 4:** Mean metabolite estimated precision (%CRLB) by group for each of four metabolites in three voxels

	*Control mean (s.d.)*	*ADHD*			P*-value*
		*Total*	*Medicated*	*Not medicated*	
		*mean (s.d.)*	*mean (s.d.)*	*mean (s.d.)*	
*Basal ganglia*					
Cho	0.095 (0.026)	0.097 (0.027)	0.089 (0.018)	0.104 (0.032)	0.279
Cr	0.071 (0.019)	0.071 (0.014)	0.068 (0.009)	0.073 (0.016)	0.581
NAA	0.100 (0.028)	0.109 (0.023)	0.106 (0.022)	0.111 (0.025)	0.393
Glx	0.097 (0.04)	0.100 (0.044)	0.086 (0.012)	0.111 (0.056)	0.194
*DLPFC*					
Cho	0.075 (0.018)	0.067 (0.011)	0.068 (0.012)	0.066 (0.011)	0.161
Cr	0.067 (0.013)	0.061 (0.008)	0.063 (0.009)	0.06 (0.008)	0.120
NAA	0.072 (0.017)	0.067 (0.012)	0.067 (0.011)	0.067 (0.013)	0.456
Glx	0.144 (0.056)	0.103 (0.033)	0.099 (0.033)	0.105 (0.034)	0.004
*Parietal cortex*					
Cho	0.089 (0.015)	0.091 (0.015)	0.093 (0.016)	0.090 (0.015)	0.654
Cr	0.066 (0.012)	0.065 (0.01)	0.068 (0.012)	0.063 (0.009)	0.511
NAA	0.072 (0.012)	0.075 (0.015)	0.076 (0.017)	0.074 (0.014)	0.671
Glx	0.092 (0.02)	0.101 (0.042)	0.095 (0.023)	0.105 (0.053)	0.488

Abbreviations: ADHD, attention-deficit hyperactivity disorder; Cho, choline; Cr, creatine; %CRLB, % Cramer–Rao Lower Bounds; DLPFC, dorsolateral predfrontal cortex; Glx, glutamate and glutamine; NAA, N-acetylaspartate.

Data are given as mean (s.d.). *P*-value: two-tailed *t*-test for analysis of variance comparing control; ADHD medicated and ADHD naive groups by %CRLB.

## Notes

GJB received honoraria for teaching from General Electric and acted as a consultant for IXICO during the course of this study. The remaining authors declare no conflict of interest. All persons designated as authors qualified for authorship. The corresponding author (JH) had full access to all of the data in the study and takes responsibility for the integrity of the data and the accuracy of the data analysis.
